# Association of Social Support and Medication Adherence in Chinese Patients with Type 2 Diabetes Mellitus

**DOI:** 10.3390/ijerph14121522

**Published:** 2017-12-06

**Authors:** Linni Gu, Shaomin Wu, Shuliang Zhao, Huixuan Zhou, Shengfa Zhang, Min Gao, Zhiyong Qu, Weijun Zhang, Donghua Tian

**Affiliations:** 1 China Institute of Health, School of Social Development and Public Policy, Beijing Normal University, 19 Xinjiekou Wai Street, Haidian District, Beijing 100875, China; gulinni@hotmail.com (L.G.); wushaominsd@163.com (S.W.); chouhuixuan@live.cn (H.Z.); zhangshengfa1988@sina.com (S.Z.); vivianhbs@126.com (M.G.); qzy@bnu.edu.cn (Z.Q.); 2 School of Public administration, Shandong Technology and Business University, 191 Binhaizhong Road, Yantai 264000, China; pingyuanzhaosan@163.com

**Keywords:** type 2 diabetes mellitus, social support, medication adherence

## Abstract

The prevalence of diabetes is steadily increasing in China. When diabetes is uncontrolled, it generates dire consequences for health and well-being. Numerous studies have shown that health outcomes were associated with social support and medication adherence. Previous study confirmed that social support was associated with medication adherence in patients with heart failure, HIV diseases, and first-episode psychosis. However, the relationship between social support and medication adherence in patients with type 2 diabetes mellitus (T2DM) is remains unclear. This study aims to examine whether social support is associated with medication adherence in patients with T2DM. This study was conducted in the First Affiliated Hospital of the General Hospital of the People’s Liberation Army (PLA). In Beijing, a systematic random sample of 412 patients with T2DM over 18 years was recruited at baseline, and demographic characteristics, clinical data and their assessment of social support were collected from medical records and self-reported questionnaires. 330 of these patients completed a self-report measure of medication adherence at the sixth month after baseline data collection. Regression analysis showed that social support presented a positive effect on medication adherence, additionally, support utilization and the subscale of social support exhibited a significantly strong influence on medication adherence in patients with T2DM. Although medication adherence was influenced by multiple factors, this finding confirmed that social support must be recognized as a core element in interventions aimed at improving in the management of patients with T2DM.

## 1. Introduction

The prevalence of diabetes is steadily increasing in China. According to a World Health Organization (WHO) report, almost of 10% of all adults in China—about 110 million people—currently live with diabetes [1]. Studies on diabetes in China reported that diabetes patients tend to have inadequate glycemic control [2]; only 41.1% of patients with type 2 diabetes mellitus (T2DM) were found to have glycated hemoglobin (HbA1c) < 7.0% (<53 mmol/mol) [3]. Previous study showed that positive health outcomes in diabetes patients were positively associated with good medication adherence [4,5,6,7]. Numerous studies on interventions to improve self-management of patients with T2DM have been carried out [8,9,10]; however, the interventions to medication adherence still need further optimization in the future [11,12]. Social support refers to a patient’s perceived and received support from his or her social network, such as familial relations, friends, neighbors, colleagues, fellow patients, or even pen friends and internet contacts to reduce psychological stress response, relieve mental stress, and improve social adaptability [13,14,15]. According to Xiao’s social support theory [15,16,17], the social support scale is composed of three dimensions: (1) subjective support, including the emotional experience and satisfaction of individuals being respected, supported and understood in society; (2) objective support, referring to the direct material assistance from social networks, or participation in communities; (3) support utilization, mainly referring to whether that individual accesses and receives the various support methods and attempts to seek support from family, relatives, friends, colleagues, and larger communities [15,16,17,18]. Previous studies demonstrated that social support was an important factor in illness recovery through the mediating mechanism of medication adherence [19,20,21,22,23]. An early study also showed that social support was associated with treatment adherence in heart failure patients through self-efficacy [24]. Another recent study in China showed that social support was a key element in the glycemic control through the work of adherence [18]. Assistance and support from families, friends, and other individuals have been connected with promoting patients’ adherence by encouraging optimism and self-esteem, buffering the stresses of being ill, reducing patient depression, and improving sick-role behavior [23,25]. Comparatively, a recent study revealed the role of social support in patients with T2DM in exploring the relationship between depressive symptoms and medication non-adherence [26]. A direct relationship between social support and medication adherence was found in patients with HIV, which pointed out that social support was particularly important because patients with HIV were easily stigmatized [27]. An earlier study explored the relationship between social support and treatment adherence in patients with non-insulin dependent diabetes mellitus in Mexico [28], which emphasized that the adherence to diet and medication were strongly associated with social support. Furthermore, some researchers studied the relationship between the different types of social support and medication adherence [29]. However, these studies did not directly examine the relationship between social support and medication adherence in patients with T2DM. Moreover, the study was not performed in China.

This study aimed to examine the relationship between social support and medication adherence in patients with T2DM. Theoretically, social support from family, friends and other social networks was expected to enhance medication adherence; that is, better social support perceived or received by a patient with T2DM and better medication adherence. Therefore, we hypothesize that social support is positively associated with medication adherence in this study.

## 2. Methods

### 2.1. Participants

This study was conducted at the department of endocrinology at First Affiliated Hospital of the General Hospital of the People's Liberation Army (PLA) in China. The sample procedures were showed in our recently published articles [30]. All participants over 18 years who were diagnosed with T2DM according to the diagnostic criteria of the 10th revision of the International Classification of Diseases (ICD-10) [31] were selected from their medication records. Those having a history of psychiatric illness and those taking anti-depressant treatment and/or using psychotropic drugs were excluded from this study. Eventually, 331 participants completed the follow-up investigation and accepted the medication adherence questionnaire at the sixth month after baseline data collection. All patients were approached by their diabetologist when they came into hospital for re-examination. The variable information at baseline and six months later in this study has been described in our previous articles [30,32].

Specifically, the demographic and socioeconomic factors, clinical characteristics and social support rate scale (SSRS) information were collected at baseline. Meanwhile, medication adherence information was also gathered six months later.

### 2.2. Ethics Statement

This study was approved by the Ethics Committee of School of Social Development and Public Policy at Beijing Normal University (BNUSSDPP2012B-0006) and the Ethics Committee of the First Affiliated Hospital of the General Hospital of PLA. All patients provided the written informed consent, and all personal information was kept confidential and reporting was made anonymous.

### 2.3. Measurements

#### 2.3.1. Demographic Information

All patients’ demographic information and socioeconomic factors were collected from the questionnaire at the investigation, which included age, gender, education level, marriage status, employment status, and insurance type.

#### 2.3.2. Clinical Characteristics

The variables including the duration of T2DM, diabetes complication, and body mass index (BMI) were gathered at baseline.

#### 2.3.3. Social Support

Social support of patients was evaluated by using the 14-item social support rate scale (SSRS), which measures objective support (i.e., actual support or material direct support, etc.), subjective support (i.e., experience or emotional support, being respected, understood or/and satisfied), and support utilization (i.e., accept help and seek help from others). It was developed by Xiao Shuiyuan in 1986, and was validated in a previous study [33]. The Cronbach’s alpha of the SSRS scale was 0.74 in this study.

#### 2.3.4. Medication Adherence

The Chinese version of the Morisky Medication Adherence Scale (MMAS-8-CN), which has been translated and cross-culturally adapted by W. Jie [34] was used to assess the medication adherence of patients with T2DM in this study [35]; the Cronbach’s alpha based on standardized items was 0.84 [32].

### 2.4. Statistical Analysis

In order to examine the relationship between social support and medication adherence after controlling the demographic and clinical variables, the flowing analytical strategies were employed. Firstly, descriptive statistics was used to present the basic characteristic of sample. Secondly, regression analysis was used to test the association between social support and the medication adherence of patients with T2DM. All the data were analyzed by using stata (16.0SE, StataCorp., College Station, TX, USA), and *p* value of <0.05 was considered statistically significant.

## 3. Result

### 3.1. Demographic Characteristic

As shown in Table 1, the mean age of the 331 participants (158 female) was 57.23 (SD = 11.43). Among of them, 241 participants demonstrated low medication adherence. For all respondents, 74.02% were married or living with their partner, and 25.98% were single (unmarried, divorced or widowed). In addition, elementary education level (≤6) applied to 11.18%, middle and high school level (6–12 years) was 36.86%, college graduate or above accounted for 51.96%. As for employment status, 58.48% were retired subjects, the highest ratio of the all subjects; employment and unemployment were at 36.36% and 5.15%, respectively. Meanwhile, participants with high medication adherence scores had higher medical reimbursement rates than those in the low scores group. The difference in basic information between the respondents and non-respondents was also compared in our previous study [32]; there were no significant differences between male and female, although the ages of the respondents were less than the non-respondents.

### 3.2. Clinical Characteristics

For this sample, more than half of the participants experienced 6–15 years duration of diabetes, 41.39% experienced less than 6 years, and 7.85% experienced more than 15 years. In addition, nearly 81% suffered from diabetes complications; the top three of the complications were cardiovascular diseases, hypertension, and abnormal blood lipid, respectively. The average number of complication for each subjects was 2.18 (SD = 1.78). The subjects with more diabetes complication were more likely to exhibit low medication adherence (t = −4.50, *p* = 0.000).

### 3.3. Social Support as a Predictor of Medication Adherence

*t* test (Table 1) was employed to examine whether a significant difference existed in variables between the two medication adherence groups. The results showed that a significant difference in term of social support scores existed between the low medication adherence group and high medication adherence group (*t* = −2.11, *p* = 0.036).

Furthermore, multiple linear regression models (Table 2) were used to explore the association between social support and medication adherence. In the first step, we found that gender, age, education level, marriage status and employment status did not account for a significant amount of medication adherence; the total adj-*R^2^* was 0.09. In the second step, the variables of insurance and clinical characteristics were introduced into the multiple linear regression model, which were combined to explain another 9% of the variance. Comparatively, social support in the third step showed a significant difference, which presented a predictor of medication adherence (β = 0.08, *p* = 0.003); this explained another 2% of variance, independently. Lastly, in order to examine the role of each subscale of social support, three subscales were also separately entered into the models. Although the adj-*R^2^* of the full model remains unchanged, the subscale of support utilization presented a significant association on medication adherence (β = 0.29, *p* = 0.011), rather than another two subscales of subjective (β = −0.02, *p* = 0.80) and objective support (β = −0.04, *p* = 0.33).

## 4. Discussion

To the best of our knowledge, this may be the first study to examine the relationship between social support and medication adherence in patients with T2DM. In this study, we examined the correlation between social support and medication adherence in patients with T2DM. The primary finding of this study confirmed the hypothesis that social support significantly influenced the medication adherence after controlling for the other variables, which is similar to the result of previous study in patients with HIV [27].

Just as in the previous studies [36,37,38,39], social support presented a positive effect on medication adherence, as well as in anxiety and depressive symptoms in various disease treatment. The study conducted in Mexico on non-insulin dependent diabetes mellitus revealed that social support acted as the main determinant of compliance with treatment [40]. Another study also found that higher scores of social support resulted in high adherence to insulin administration in women with gestational diabetes [41]. Similarly, the studies conducted by other researchers on the relationship between social support and medication adherence in patients with HIV also obtained consistent results [42,43,44]. Nearly all of them confirmed a positive association between social support and medication adherence. Additionally, Jean M. Breny Bontempi employed a qualitative study to explore the importance of social support in HIV medication adherence programs, namely the patients with HIV need more social support because HIV therapy was very complex [44]. A similar study, conducted in A South Africa, also showed that social support played a critical role in adherence to therapy for those discriminatory patients with HIV [41]. Of course, there are different opinions. An earlier study revealed that the effect of social support on medication adherence did not persist over time, and that the changes of social support may result in adherence alteration [45].

Examining the effect of social support on medication adherence in patients with T2DM was a main contribution of this study. The mechanism of this association still needs to be further explored. Previous studies have also obtained some results; for example, a study of HIV-infected men revealed that social support influenced medication adherence indirectly, while current anxious and depressive symptoms mediated the relationship between general social support and recent self-reported medication adherence in HIV-infected population of MSM (sex with men) [46]. Similarly, another study also found that the relationship between social support and adherence was mediated by negative effects, spirituality, and self-efficacy [47]. Furthermore, an early study indicated that the characteristics of social support played a role in the relationship between social support and medication adherence [27]. Therefore, questions about whether mediating variables exist between social support and medication adherence in patients with T2DM were especially important and should be further explored in the future.

It is well known that medication adherence is crucial to patients with T2DM in the controlling of blood glucose. Higher rates of medication adherence can reduce the risk of known complications [42]. It is also easily to understand that medication adherence, as self-management, can significantly influence glycemic control. Poor medication adherence would result in bad glycemic control of the blood glucose level. There were also several factors, identified by previous studies, which influenced the medication adherence of patients with diabetes, such as multiple medicine taking, social and economic-related factors, and therapy-related factors [40].

Perceived and received social support is becoming important in the management of patients with T2DM; for one thing, it was related to the progress of a variety of diabetes complications [32,41,48,49]. Several studies suggested that individuals with more supportive families or friends recovered faster than those who were less socially integrated [43,44]. Lack of social support resulted in maladaptive responses to diabetes, further weakening the fighting spirit of diabetes patients. Additionally, social support was always related to subjective well-being [45,50]; once patients perceived and received social support from outsiders, they feel they are concerned about, accepted, and appreciated and cared for by other individuals, and their subjective well-being will be quickly increased; they would therefore take a positive attitude to their chronic diseases. Generally speaking, social support comes from siblings, partners and confidantes who influence health-related decision-making [27]; patients who gain their support will feel psychologic comfort and deal with health problems in a positive way. Besides this, social support may bring material assistance to individuals sometimes. The cost of disease treatment is very high, and getting support from other individuals may help in relieving patients’ financial burdens, and further improve patient’s medication adherence.

There are different opinions about the categorization of social support in previous studies. For example, some researchers categorized social support as emotional support, practical support, behavioral support and so on [29,51]. Different categorizations may generate different result in the statistical analysis. In this study, we found that support utilization was identified as a determined factor in three dimensions; this means that utilization from social support determined whether individuals had actual contact with other persons and social groups. There were also different findings; several previous studies showed that perception of social support was more important than utilization [41,52,53]. However, Isao Fukunishi, with other researchers, suggested that utilization is equally important as perception [38]. If patients accepted support from other individuals or groups, such as respect, understanding and support, they would be boosted a positive mood, as well as model desired actions by providing information and examples, which may affect self-efficacy beliefs [54], and in turn influence their medication adherence. Only when individuals received support from others and then transferred them into activities could they adhere to medication. Likewise, seeking help from others can relieve the harmful influence of negative feelings on the process of diabetes treatment. Altogether, in order to improve the management of patients with T2DM, variable social support, as an environmental factor, and its effect on medication adherence and health outcomes should be worthy of further study.

## 5. Limitations

Some limitations should be recognized here. Firstly, the sample size was small and the sample was collected from a general hospital, the results may not fully be extrapolated to the whole population with T2DM. Future studies should include a large sample size so that the complex dynamics between social support and medication adherence could be examined. Secondly, the study relied on self-reported measurement of social support and medication adherence, which might result in the self-reported bias. Further study should be conducted to investigate the relationship between social support and objective medication adherence.

## 6. Conclusions

To our knowledge, this may be the first study to explore the relationship between social support and medication adherence in patients with T2DM in China. The findings indicated that social support acts as a crucial part in improving medication adherence in diabetes patients. This means that patients with T2DM need to open their minds by gaining help from friends, relatives and other organizations. In the treatment process, family members play a major role, which requires them to establish a supportive environment and amend supportive behavior in improving patients’ medication adherence, such as reminding patients to take medicine on time, supervising a healthy diabetic diet, etc. The study also found that utilization support is the core element in medication adherence in patients with T2DM. That is to say, actively accepting and seeking help from outside may maximize the treatment outcome and relieve the negative effects of disease, such as depression, anxiety and other mental illness. These findings may help patients with T2DM to improve the self-management of disease under the help of other individuals and groups. Likewise, these findings contributed to a growing body of further studies on the relationship between social support and medication adherence.

## Figures and Tables

**Table 1:** Sample characteristics.

Variables	All Patients (*N* = 331)	Low Adherence (*N* = 241)	High Adherence (*N* = 90)	*p* Value
	*N*, %	*N*, %	*N*, %	
**Sex**				Phi = 1.00, *p* = 0.318
Female	158 (47.73)	111 (46.06)	47 (52.22)	
Male	173 (52.27)	130 (53.94)	43 (47.78)	
**Age**	57.23 ± 11.43	56.88 ± 11.41	58.17 ± 11.50	*t* = −0.91, *p* = 0.363
**Marital status**				Phi = 0.21, *p* = 0.649
Single	86 (25.98)	61 (25.31)	25 (27.78)	
Married	245 (74.02)	180 (74.69)	65 (72.22)	
**Family income**	9.85 ± 10.61	10.09 ± 11.84	9.23 ± 6.26	*t* = 0.65, *p* = 0.517
**Education level**				Cramer’s V = 0.07, *p* = 0.483
≤6 years	37 (11.18)	30 (12.45)	7 (7.78)	
6–12 years	122 (36.86)	88 (36.51)	34 (37.78)	
≥12 years	172 (51.96)	123 (51.04)	49 (54.44)	
**Employment status**				Cramer’s V = 0.12, *p* = 0.079
Employed	120 (36.36)	96 (40)	24 (26.67)	
Unemployed	17 (5.15)	12 (5)	5 (5.56)	
Retired	193 (58.48)	132 (55)	61 (67.78)	
**Insurance type**				Cramer’s V = 0.15, *p* = 0.070
GIS	105 (31.82)	68 (28.33)	37 (41.11)	
UEMI	95 (28.79)	73 (30.42)	22 (24.44)	
NCMS	77 (23.33)	55 (22.92)	22 (24.44)	
Other insurance	53 (16.06)	44 (18.33)	9 (10)	
**Reimbursement ratio:**	77.25 ± 24.39	74.85 ± 26.14	83.66 ± 17.54	*t* = −3.52, *p* = 0.001
**BMI**	24.99 ± 3.16	25.17 ± 3.31	24.52 ± 2.70	*t* = 1.68, *p* = 0.094
**Duration of DM**				Cramer’s V = 0.03, *p* = 0.871
≤5 years	137 (41.39)	100 (41.49)	37 (41.11)	
6–15 years	168 (50.76)	121 (50.21)	47 (52.22)	
≥16 years	26 (7.85)	20 (8.30)	6 (6.67)	
**No of Diabetes complications**	2.18 ± 1.78	2.42 ± 1.83	1.54 ± 1.47	*t* = −4.50, *p* = 0.000
**Social support**	31.74 ± 5.28	31.36 ± 5.23	32.73 ± 5.31	*t* = −2.11, *p* = 0.036
Subjective support	16.49 ± 3.45	16.22 ± 3.28	17.21 ± 3.80	*t* = −2.34, *p* = 0.020
Objective support	8.08 ± 1.93	8.05 ± 1.99	8.14 ± 1.76	*t* = −0.40, *p* = 0.692
Support utilization	7.15 ± 1.36	7.07 ± 1.34	7.38 ± 1.40	*t* = −1.85, *p* = 0.065

**Table 2:** Hierarchical multiple linear regression predicting medical adherence.

Variables	Step 1	Step 2	Step 3	Step 4
	β (95% CI)	β (95% CI)	β (95% CI)	β (95% CI)
Demographic Factors				
Age	−0.00 (−0.03, 0.03)	−0.00 (−0.03, 0.03)	−0.01 (−0.04, 0.02)	−0.01 (−0.03, 0.02)
Gender				
Female				
Male	−0.81 (−1.34, −0.29) **	−0.57 (−1.07, −0.06) *	−0.52 (−1.03, −0.02) *	−0.43 (−0.95, 0.08)
Education level				
Primary school or lower				
Middle and high school	0.72 (−0.16, 1.59)	0.44 (−0.42, 1.31)	0.49 (−0.37, 1.35)	0.63 (−0.24, 1.51)
College and above	1.31 (0.41, 2.20) **	0.81 (−0.09, 1.72)	0.88 (−0.01, 1.78)	0.96 (0.06, 1.86) *
Marriage status				
Single				
Married	−0.45 (−1.04, −0.14)	−0.63 (−1.22, −0.04) *	−0.81 (−1.42, −0.20) **	−0.81 (−1.42, −0.20) **
Employment status				
Employed				
Unemployed	1.66 (0.46, 2.86) **	1.60 (0.41, 2.79) **	1.58 (0.41, 2.76) **	1.57 (0.38, 2.75) **
Retired	1.20 (0.50, 1.89) ***	0.90 (0.20, 1.59) *	0.97 (0.28, 1.66) **	0.90 (0.21, 1.59) *
income	−0.01 (−0.03, 0.02)	0.01 (−0.02, 0.03)	0.01 (−0.02, 0.03)	0.00 (−0.02, 0.03)
Insurance				
GIS				
UEMI		−0.79 (−1.43, −0.15) *	−0.68 (−1.32, −0.05) *	−0.57 (−1.22, 0.07)
NCMS		−0.16 (−0.86, 0.54)	0.09 (−0.62, 0.79)	0.20 (−0.51, 0.95)
Other insurance		0.07 (−1.13, 1.27)	0.44 (−0.77, 1.64)	0.62 (−0.60, 1.84)
Reimbursement ratio		0.03 (0.01, 0.04) ***	0.03 (0.01, 0.04) ***	0.03 (0.01, 0.05) ***
BMI		−0.02 (−0.10, 0.06)	−0.04 (−0.12, 0.04)	−0.02 (−0.10, 0.07)
History of diabetes				
No				
yes		0.06 (−0.46, 0.59)	−0.03 (−0.55, 0.49)	0.00 (−0.52, 0.52)
Duration of diabetes in years		0.07 (0.01, 0.12) *	0.07 (0.01, 0.12) *	0.06 (0.01, 0.11) *
Diabetes complication		−0.34 (−0.50, −0.18) ***	−0.30 (−0.47, −0.14) ***	−0.29 (−0.46, −0.12)
Social support (total score)			0.08 (0.03, 0.13) **	
Social support (three waves)				
Subjective support				0.02 (−0.13, 0.17)
Objective support				0.04 (−0.04, 0.13)
Support utilization				0.29 (0.07, 0.51) *
Cons	4.59 (2.73, 6.46) ***	4.07 (1.25, 6.89) **	2.00 (−1.12, 5.10)	0.56 (−2.89, 3.99)
Adj-*R^2^*	0.09	0.18	0.2	0.2
Total number	330	330	328	328

* 0.01 < *p* ≤ 0.05, ** 0.001 < *p* ≤ 0.01, *** *p* ≤ 0.001.

## References

[1] World Health Organization Rate of diabetes in China “explosive”. http://www.wpro.who.int/china/mediacentre/releases/2016/20160406/en/.

[2] Xu Y., Wang L., He J., Bi Y., Li M., Wang T., Wang L., Jiang Y., Dai M., Lu J. (2013). Prevalence and control of diabetes in Chinese adults. J. Am. Med. Assoc. USA.

[3] Pan C., Yang W., Jia W., Weng J., Tian H. (2009). Management of Chinese patients with type 2 diabetes, 1998–2006: The Diabcare-China Surveys. Curr. Med. Res. Opin..

[4] Harith K.A.-Q., Sulaiman S.A., Hassali M.A., Shafie A.A., Sundram S., Al-Nuri R., Saleem F. (2011). Diabetes knowledge, medication adherence and glycemic control among patients with type 2 diabetes. Int. J. Clin. Pharm..

[5] Cummings D.M., Lutes L., Littlewood K., DiNatale E., Hambidge B., Schulman K., Morisky D.E. (2014). Regimen-Related Distress, Medication Adherence, and Glycemic Control in Rural African American Women With Type 2 Diabetes Mellitus. Ann. Pharmacother..

[6] Feldman B.S., Cohen-Stavi C.J., Leibowitz M., Hoshen M.B., Singer S.R., Bitterman H., Lieberman N., Balicer R.D. (2014). Defining the role of medication adherence in poor glycemic control among a general adult population with diabetes. PLoS ONE.

[7] Virdi N., Daskiran M., Nigam S., Kozma C., Raja P. (2012). The association of self-monitoring of blood glucose use with medication adherence and glycemic control in patients with type 2 diabetes initiating non-insulin treatment. Diabetes Technol. Ther..

[8] Glasgow R.E., Barrera M., Mckay H.G., Boles S.M. (1999). Social support, self-management, and quality of life among participants in an internet-based diabetes support program: A multi-dimensional investigation. Cyberpsychol. Behav..

[9] Hunt C.W., Wilder B., Steele M.M., Grant J.S., Pryor E.R., Moneyham L. (2012). Relationships among self-efficacy, social support, social problem solving, and self-management in a rural sample living with type 2 diabetes mellitus. Res. Theor. Nurs. Pract..

[10] Hunt C.W. (2012). Relationships among Self-Efficacy, Social Support, Social Problem-Solving, and Self-Management Behaviors of People Living with Type 2 Diabetes in Rural Alabama. Ph.D. Thesis.

[11] Vervloet M., Dijk L.V., Santen-Reestman J., Vlijmen B.V., Wingerden P.V., Bouvy M.L., Bakker D.H.D. (2012). SMS reminders improve adherence to oral medication in type 2 diabetes patients who are real time electronically monitored. Int. J. Med. Inf..

[12] Lin L.K., Sun Y., Heng B.H., Chew D., Chong P.N. (2017). Medication adherence and glycemic control among newly diagnosed diabetes patients. Bmj Open Diabetes Res. Care.

[13] Van Dam H.A., van der Horst F.G., Knoops L., Ryckman R.M., Crebolder H.F., van den Borne B.H. (2005). Social support in diabetes: A systematic review of controlled intervention studies. Patient Educ. Couns..

[14] Xiao S.Y. (1994). Theoretical foundation and research application about the social support rating scale. J. Clin. Psychiatry Med..

[15] Liu J., Li F., Liang Y. (2008). Investigation of reliability and validity of the social support scale. J. Xinjiang Med. Univ..

[16] Xu L., Jiang X., Liao C. (2009). Effects of Social Support and Coping Style on Quality of Life in Heart Transplantation Recipients. J. Nurs. Sci..

[17] Cheng Y., Liu C., Mao C., Qian J., Liu K., Ke G. (2008). Social support plays a role in depression in Parkinson’s disease: A cross-section study in a Chinese cohort. Parkinsonism Relat. Disord..

[18] Shao Y., Liang L., Shi L., Wan C., Yu S. (2017). The Effect of Social Support on Glycemic Control in Patients with Type 2 Diabetes Mellitus: The Mediating Roles of Self-Efficacy and Adherence. J. Diabetes Res..

[19] Schatz P.E. (1988). An evaluation of the components of compliance in patients with diabetes. J. Am. Diet. Assoc..

[20] DiMatteo M.R., Hays R.D. (1981). Social support and serious illness. Soc. Netw. Soc. Support.

[21] Helgeson V.S., Cohen S. (1996). Social support and adjustment to cancer: Reconciling descriptive, correlational, and intervention research. Health Psychol..

[22] Uchino B.N., Cacioppo J.T., Kiecolt-Glaser J.K. (1996). The relationship between social support and physiological processes: A review with emphasis on underlying mechanisms and implications for health. Psychol. Bull..

[23] Wallston B.S., Alagna S.W., DeVellis B.M., DeVellis R.F. (1983). Social support and physical health. Health Psychol..

[24] Maeda U., Shen B.-J., Schwarz E.R., Farrell K.A., Mallon S. (2013). Self-Efficacy Mediates the Associations of Social Support and Depression;with Treatment Adherence in Heart Failure Patients. Int. J. Behav. Med..

[25] Shumaker S.A., Hill D.R. (1991). Gender differences in social support and physical health. Health Psychol..

[26] Osborn C.Y., Egede L.E. (2012). The Relationship between Depressive Symptoms and Medication Non-Adherence in Type 2 Diabetes: The Role of Social Support. Gen. Hosp. Psychiatry.

[27] Ncama B.P., Mcinerney P.A., Bhengu B.R., Corless I.B., Wantland D.J., Nicholas P.K., Mcgibbon C.A., Davis S.M. (2008). Social support and medication adherence in HIV disease in KwaZulu-Natal, South Africa. Int. J. Nurs. Stud..

[28] Garaysevilla M.E., Nava L.E., Malacara J.M., Huerta R., de León J.D., Mena A., Fajardo M.E. (1995). Adherence to treatment and social support in patients with non-insulin dependent diabetes mellitus. J. Diabetes Its Complicat..

[29] Scheurer D., Choudhry N., Swanton K.A., Matlin O., Shrank W. (2012). Association between different types of social support and medication adherence. Am. J. Manag. Care.

[30] Zhang W., Xu H., Zhao S., Yin S., Wang X., Guo J., Zhang S., Zhou H., Wang F., Gu L. (2015). Prevalence and influencing factors of co-morbid depression in patients with type 2 diabetes mellitus: A General Hospital based study. Diabetol. Metab. Syndr..

[31] Alberti K.G., Zimmet P.D. (1998). Diagnosis and classification of diabetes mellitus and its complications. Part 1: Diagnosis and classification of diabetes mellitus provisional report of a WHO consultation. Diabet. Med..

[32] Li X., Zhang S., Xu H., Tang X., Zhou H., Yuan J., Wang X., Qu Z., Wang F., Zhu H. (2016). Type D Personality Predicts Poor Medication Adherence in Chinese Patients with Type 2 Diabetes Mellitus: A Six-Month Follow-Up Study. PLoS ONE.

[33] Zheng Y.P., Wei L., Goa L.G., Zhang G.C., Wong C.G. (1988). Applicability of the Chinese Beck Depression Inventory. Compr. Psychiatry.

[34] Jie W., Yongzhen M., Rongwen B. (2013). Evaluation of reliability and validity of application of the Chinese version of 8-item Morisky Medication Adherence Scale in patients with type 2 diabetes. Chin. J. Diabetes.

[35] Wang J., Bian R.W., Mo Y.Z. (2013). Validation of the Chinese version of the eight-item Morisky medication adherence scale in patients with type 2 diabetes mellitus. J. Clin. Gerontol. Geriatr..

[36] Johnson V.R., Jacobson K.L., Gazmararian J.A., Blake S.C. (2010). Does social support help limited-literacy patients with medication adherence? A mixed methods study of patients in the Pharmacy Intervention for Limited Literacy (PILL) study. Patient Educ. Couns..

[37] Bontempi J.M.B., Burleson L., Lopez M.H. (2004). HIV Medication Adherence Programs: The Importance of Social Support. J. Commun. Health Nurs..

[38] Fukunishi I., Horikawa N., Yamazaki T., Shirasaka K., Kanno K., Akimoto M. (1998). Perception and utilization of social support in diabetic control. Diabetes Res. Clin. Pract..

[39] Vyavaharkar M., Moneyham L., Tavakoli A., Phillips K.D., Murdaugh C., Jackson K., Meding G. (2007). Social support, coping, and medication adherence among HIV-positive women with depression living in rural areas of the southeastern United States. AIDS Patient Care STDS.

[40] Marzec L.N., Maddox T.M. (2013). Medication adherence in patients with diabetes and dyslipidemia: Associated factors and strategies for improvement. Curr. Cardiol. Rep..

[41] King K.B. (1997). Psychologic and social aspects of cardiovascular disease. Ann. Behav. Med..

[42] Buysman E.K., Fang L., Hammer M., Langer J. (2015). Impact of Medication Adherence and Persistence on Clinical and Economic Outcomes in Patients with Type 2 Diabetes Treated with Liraglutide: A Retrospective Cohort Study. Adv. Ther..

[43] Hogan B.E., Linden W., Najarian B. (2002). Social support interventions: Do they work?. Clin. Psychol. Rev..

[44] Rhodes J.E. (2004). Family, friends, and community: The role of social support in promoting health. Clin. Handb. Health Psychol..

[45] Kahn J.H., Hessling R.M., Russell D.W. (2003). Social support, health, and well-being among the elderly: What is the role of negative affectivity?. Personal. Individ. Differ..

[46] Woodward E.N., Pantalone D.W. (2012). The Role of Social Support and Negative Affect in Medication Adherence for HIV-Infected Men Who Have Sex With Men. J. Assoc. Nurses AIDS Care.

[47] Simoni J.M., Frick P.A., Huang B. (2006). A longitudinal evaluation of a social support model of medication adherence among HIV-positive men and women on antiretroviral therapy. Health Psychol..

[48] Sayal K., Checkley S., Rees M., Jacobs C., Harris T., Papadopoulos A., Poon L. (2002). Effects of social support during weekend leave on cortisol and depression ratings: A pilot study. J. Affect. Disord..

[49] Heckman T.G. (2003). The chronic illness quality of life (CIQOL) model: Explaining life satisfaction in people living with HIV disease. Health Psychol..

[50] Karademas E.C. (2006). Self-efficacy, social support and well-being: The mediating role of optimism. Personal. Individ. Differ..

[51] Kanbara S., Taniguchi H.M., Wang D., Takaki J., Yajima Y., Naruse F., Kojima S., Sauriasari R., Ogino K. (2008). Social support, self-efficacy and psychological stress responses among outpatients with diabetes in Yogyakarta, Indonesia. Diabetes Res. Clin. Pract..

[52] Griffith L.S., Field B.J., Lustman P.J. (1990). Life stress and social support in diabetes: Association with glycemic control. Int. J. Psychol. Med..

[53] Schwartz L.S., Coulson L.R., Toovy D., Lyons J.S., Flaherty J.A. (1991). A biopsychosocial treatment approach to the management of diabetes mellitus. Gen. Hosp. Psychiatry.

[54] Bandura A. (1997). Self-efficacy: The exercise of control. J. Cogn. Psychother..

